# Breeding schemes for intervertebral disc disease in dachshunds: Is disc calcification score preferable to genotyping of the *FGF4* retrogene insertion on CFA12?

**DOI:** 10.1186/s40575-020-00096-6

**Published:** 2020-12-01

**Authors:** Camilla Sichlau Bruun, Charlotte Bruun, Tine Marx, Helle Friis Proschowsky, Merete Fredholm

**Affiliations:** 1 Department of Veterinary and Animal Sciences, University of Copenhagen, 1870 Frederiksberg C, Denmark; 2 Hørsholm Hestepraksis, 3480 Fredensborg, Denmark; 3 Abild Dyreklinik, 8220 Brabrand, Denmark; 4 The Danish Kennel Club, 2680 Solrød Strand, Denmark

**Keywords:** *FGF4* retrogene insertion, Intervertebral disc herniation, Disc calcification, Dachshund, DNA test, Breeding program, IVDD, IVDH

## Abstract

**Background:**

Approximately every fifth Dachshund is affected by disc herniation - a painful, hereditary condition which is typically preceded by disc calcification. Therefore, the selection of dogs suitable for breeding can be based on radiographic examination of calcification status.

Recently, an insertion of an *FGF4* retrogene on CFA12 has been identified and associated with the risk of developing disc herniation in chondrodystrophic breeds and a DNA test is now offered.

In this study we investigate the incidence of disc herniation in the smooth-haired, long-haired and wire- haired Dachshund populations. We also evaluate and compare the accuracy of the two breeding schemes predicting the risk of disc herniation: the DNA test and the radiography based scheme.

**Results:**

The overall incidence of disc herniation in Danish Dachshunds was 18% and no significant difference was found between the long-haired (17%), smooth-haired (22%) and wire-haired (16%) populations (*p* > 0.05). We found a significant association (*p* <  0.0001) between calcification status and the risk of disc herniation with a relative risk of 14.78. Using calcification status (≥ 5 or <  5 calcifications) as a risk indicator has a sensitivity of 0.79 and a specificity of 0.91.

A significant association between the *FGF4* retrogene insertion and the disc calcification status was found in the wire-haired population (*p* <  0.0001) where the DNA test has a sensitivity of 1.0 and a specificity of 0.14. In the long- and smooth-haired populations no association was found (*p* > 0.05) and here the insertion allele was almost fixed.

**Conclusion:**

Our results show that the *FGF4* retrogene insertion on CFA12 is not a valid risk indicator on its own. Relying on the DNA test will have an irreversible effect on the Dachshund breed excluding almost all dogs from breeding. Thus, using calcification status remains the most reliable breeding scheme for disc herniation in Dachshunds.

**Supplementary Information:**

The online version contains supplementary material available at 10.1186/s40575-020-00096-6.

## Plain English summary

Short-legged dogs are prone to develop intervertebral disc herniation (IVDH). Approximately every fifth Dachshund suffers from this painful disease. The high incidence was confirmed in our study, and the incidence seems to be the same for the three hair variants (smooth-, long- and wire-haired). The disease starts with minor changes and calcifications in the nucleus of the cushion-like discs placed between the bones in the spine. The changed nucleus may ultimately cause disruption of the surrounding connective tissue and herniate resulting in back pain.

The tendency to develop calcifications is hereditary and the more calcifications, the greater the risk of developing IVDH. Radiographic examination of the back can reveal the number of calcifications and therefore the risk of DH can be estimated. Hence, radiographic back examination is used in the Danish Dachshund breeding scheme to reduce the incidence of IVDH. Dogs with ≥5 calcifications are excluded from breeding.

A group of researchers has recently discovered a mutation that they claim entails a risk of IVDH. A DNA test is being offered revealing whether a dog has the mutation or not.

We have compared the two methods of predicting the risk of IVDH: radiographic examination and DNA test. Dogs with ≥5 calcifications have a 14 times greater risk of developing IVDH compared to dogs with < 5 calcifications. Among the dogs who develop IVDH, 21% have < 5 calcifications indicating that environmental or other genetic factors play a role for the development of the disease. Only 9% of dogs who remain healthy have ≥5 calcifications.

The association between the mutation and number of calcifications is not as evident. Almost all the smooth- and long-haired Dachshunds have the mutation irrespective of the number of calcifications. Among the wire-haired, all dogs with many calcifications also have the mutation. However, most dogs with few calcifications have the mutation too. So if the DNA test is used in the breeding scheme, almost all dogs will be excluded.

Therefore, at present the only possible breeding scheme for reducing the incidence of IVDH in Dachshunds is a scheme relying on calcification scores.

## Background

Chondrodystrophic dog breeds, including Dachshunds, are characterized by disproportionate dwarfism owing to their inherent failure of normal endochondral ossification. Several of these breeds are prone to early onset intervertebral disc disease (IVDD) often leading to intervertebral disc herniation (IVDH), a painful debilitating condition, which in many cases calls for surgery or even euthanasia. Among dog breeds Dachshunds have the highest incidence of IVDH [1, 2] with varying estimates reported (19% [3], 31% [4], 15.9% [5]). Several attempts have been made to unravel the genetic background of IVDD in order to establish an effective breeding scheme. A new DNA test is available that claims to reveal the genetic risk for developing IVDH [6]. In this study we assess the incidence of IVDH in Dachshunds and evaluate the accuracy of two breeding schemes relying on disc calcification scores and the DNA test, respectively.

Due to the breed history we have evaluated each of the three hair variants separately. The Dachshund breed was established in Germany probably around the sixteenth century. The original Dachshunds were smooth-haired and the long- and wire-haired variants were established by crossing in spaniels and terriers, respectively, in the late nineteenth century [7]. Even if the three variants belong to the same breed they are bred separately and are therefore to some extent genetically divergent as shown by Mogensen et al. [8].

IVDD is a continuum of processes leading to necrosis and calcification of the centrally placed nucleus pulposus and weakening of the surrounding annulus fibrosus. This degeneration goes on in all discs simultaneously [9–11]. The calcified disc material may, however, disappear - probably due to phagocytic resorption [12]. Ultimately the degenerative processes may result in rupture of the annulus fibrosus and disc herniation referred to as “Hansen type 1” [10, 11, 13].

IVDD has a multifactorial etiology involving both genetic and environmental components [3] also reflected in the continuous variation of disease stages. With few exceptions disc herniation is always preceded by calcification which has an estimated heritability of 0.6–0.87 [14, 15]. The risk of developing IVDH can therefore be estimated by evaluating the number of calcified discs – a strategy used in Dachshund breeding programs in some countries. The number of calcified discs reaches a maximum at 24–27 months and radiographic examination is thus recommended at 24–30 months of age [4, 16]. In the Danish Dachshund Club the breeding program has been based on radiographic back examination at the age of 24 to 48 months since 2003, and only dogs with less than 5 calcifications are accepted for breeding. In addition, estimated breeding values (EBV) are calculated. The breed average is set to 100 and an average EBV of the parents of 100 or above is recommended.

To identify one or more causative mutations Mogensen et al. [8] performed a genome wide association study (GWAS) with cases and controls representing all three Dachshund hair variants (smooth-, wire- and long-haired). Stratification analysis revealed that the three hair variants belonged to different clusters and they were therefore analyzed separately. The most significant association was identified in the wire-haired cluster on *Canis familiaris* chromosome (CFA) 12 in the region 36.8 to 38.6 Mb (CanFam2) [8]. However, no causative variants were identified. Three single nucleotide polymorphisms (SNPs) in protein coding genes in the candidate region showed significant association with calcification status in the wire-haired whereas the smooth- and long-haired dogs were fixed for the variants associated with IVDD in the wire-haired dogs [17].

Another GWAS across breeds pointed at the same region on CFA12 where a fibroblast growth factor 4 (FGF4) retrogene insertion (*Ins*) was found to be associated with IVDD [18]. A DNA test revealing the risk of developing IVDH based on the *Ins* genotype is now offered at the UC Davis Veterinary Genetics Laboratory [6]. The mutation is reported to have an additive effect on disc calcification and a dominant effect on the risk of IVDH [19]. In the studies conducted by Brown et al. [18] and Batcher et al. [19] all three hair variants were treated as one.

Here we report and compare the incidence of IVDH in the three Dachshund hair variants. We also evaluate and compare the accuracy of a breeding program based on disc calcification scores and a scheme based on the DNA test. As opposed to radiographic examination a valid DNA test would allow for an earlier, cheaper and easier selection of dogs for breeding.

## Materials and methods

### Incidence study

In 2014, a questionnaire (see Additional file 1) was sent to owners of 267 wire haired, 254 long haired and 236 smooth haired Dachshunds randomly selected among Dachshunds born in 2002–2004 and registered in the Danish Kennel Club. Contact information was provided by the Danish Kennel Club. Thus, all dogs were ≥ 9 years of age or dead. Owners were asked about clinical signs of IVDH (signs of back pain, reluctance to walk or jump etc.), whether the dog had been diagnosed with IVDH by a veterinarian and - if relevant - cause of death. Based on the answers the dogs were divided in three non-overlapping categories: “free of clinical signs”, “diagnosed with IVDH” and “IVDH clinical signs” (no diagnosis).

Dogs were excluded if the questionnaire was incompletely filled out or if they were dead or euthanized for other reasons than IVDH before 4 years of age.

The incidence was calculated as the number of dogs diagnosed with IVDH/number of dogs at the beginning of the study. To analyze whether the incidence was different among the three hair variants a χ2 test was performed using SciStat® [20] and a significance level of 0.05. Only the two groups “free of clinical signs” and “diagnosed with IVDH” were included in the χ2 test.

### Follow up study

The same questionnaire (see Additional file 1) was sent to (all) 154 owners of Dachshunds registered in the Danish Kennel Club and radiographically examined at the age of 24–48 months in 2004–2006. Contact information was provided by the Danish Kennel Club. Again, based on the answers the dogs were divided in three non-overlapping categories: “free of clinical signs”, “diagnosed with IVDH” and “IVDH clinical signs” (no diagnosis). Information on number of calcifications and EBVs calculated April 1. 2014 for each dog was provided by the Danish Kennel Club.

EBV are calculated using a best linear unbiased prediction (BLUP) animal model. The model includes gender, year of screening, hair variant and a fixed regression on age at screening. The breed average is set to 100 and the standard deviation is 15.

To analyze the association of calcification number (< 5 and ≥ 5), EBV (< 100 and ≥ 100) and IVDH, χ2, relative risk (RR) and odds ratio (OR) were calculated using SciStat® [20]. A significance level of 0.05 was chosen for the χ2 test and confidence intervals were estimated for the RR and OR. Only the two groups “free of clinical signs” and “diagnosed with IVDH” were included in the analysis.

The sensitivity of the radiographic examination was calculated as number of dogs with ≥5 calcifications and diagnosed with IVDH or with IVDH clinical signs/number of dogs diagnosed with IVDH or with IVDH clinical signs.

The specificity was calculated as number of dogs free of clinical signs and <  5 calcifications/ number of dogs free of clinical signs.

### Genotyping

Ethylene-diamine-tetra-acetic (EDTA) stabilized blood samples were collected from 151 Dachshunds by veterinarians in relation to radiographic back examination at the age of 24–48 months. All dogs were registered in the Danish Kennel Club and the number of disc calcifications was reported by a trained veterinary radiologist. DNA was extracted using a salt precipitation method [21].

The dogs were genotyped for the *FGF4* retrogene insertion on CFA12 according to Brown et al. [18] using a three primer PCR: a forward and reverse primer pair flanking the insert locus (5-‘ACAGCTGGCATGGTCAGTTA-3’ and 5′-TGCTGTAGATTTTGAGGTGTCTT-3′) resulting in a 333 bp PCR fragment representing the normal allele (*N*), and an additional forward primer annealing within the insert (5′-GTCCGTGCGGTGAAATAAAA-3′) resulting in a 654 bp fragment representing the *FGF4* retrogene insertion allele (*Ins*). Since, in our hands, the level of amplification of the 654 bp fragment was low, dogs apparently homozygous of the normal allele (i.e. no insertion, *N*/*N*) were in addition genotyped separately using the insert specific forward and the reverse primer pair alone.

### Association

The dogs were divided in two groups according to the number of calcifications (≥ 5 calcifications or IVDH and < 5 calcifications). Association between the *Ins* allele and number of calcifications was analyzed separately for each hair variant using a χ2 test and a significance level of 0.05.

Calculation of χ2 for the long-haired was performed after adding a constant of 1 to all observations to mask out zero columns due to (non-structural) sampling zeros.

The frequency of the *Ins* allele was calculated as the number of *Ins* alleles/total number of alleles.

DNA test sensitivity was calculated as number of dogs with an *Ins*/− genotype and ≥ 5 calcifications/number of dogs with ≥5 calcifications.

The specificity was calculated as number of dogs with the genotype *N*/*N* and < 5 calcifications/ number of dogs with < 5 calcifications.

## Results

### Incidence study

Of the 560 returned questionnaires 503 comprised sufficient information to be included in the study. They represented 175 wire-haired, 175 long-haired and 153 smooth-haired Dachshunds. The results are presented in Table 1.

**Table 1:** Incidence of IVDH and IVDH clinical signs in 9 (or more) years old Dachshunds

Hair variant	Diagnosed with IVDH	IVDH clinical signs	Free of clinical signs	Total	IVDH or IVDH clinical signs
Wire-haired	28 (16*%*)	15 (9*%*)	132 (75*%*)	175 (100*%*)	43 (25*%*)
Long-haired	30 (17*%*)	13 (7*%*)	132 (75*%*)	175 (100*%*)	43 (25*%*)
Smooth-haired	33 (22*%*)	11 (7*%*)	109 (71*%*)	153 (100*%*)	44 (29*%*)
Total	91 (18*%*)	39 (8*%*)	373 (74*%*)	503 (100*%*)	130 (26*%*)

The incidence of IVDH was estimated at 16.0% (wire-haired), 17.43% (long-haired) and 21.57% (smooth-haired). There was no significant difference in IVDH incidence between hair variants. *P*-values were 0.31 (smooth- vs. long-haired), 0.22 (smooth- vs. wire-haired) and 0.81 (long- vs. wire-haired).

Among the dogs diagnosed with IVDH 33% had been euthanized due to this condition.

### Follow up study

Of the 122 returned questionnaires 117 comprised all the needed information. The results are presented in Table 2.

**Table 2:** IVDH and IVDH clinical signs in dogs with ≥5 and < 5 calcifications

Number of calcifications	Diagnosed with IVDH	IVDH clinical signs	Free of clinical signs	Total
≥ 5	19	4	8	31
< 5	4	2	80	86
Total	23	6	88	117

The number of dogs in each calcification group (0–12) and their back status is shown in Additional file 2.

The test sensitivity was (19 + 4)/(23 + 6) = 0.79 and the test specificity was 80/88 = 0.91.

The risk of developing IVDH was significantly different for the two calcification groups**:** χ2 = 53.5 (*p* < 0.0001); RR = 14.78 (confidence interval: 5.5064 to 39.6595); OR = 47.5 (confidence interval: 12.94 to 174.35).

As seen in Table 3 the risk of developing IVDH was significantly different for the two EBV groups: χ2 = 30.74, *p* < 0.0001, RR = 15.4 (3.7974 to 62.4536); OR = 28.0 (confidence interval: 6.0970 to 128.5881).

**Table 3:** IVDH and clinical signs of IVDH in dogs with EBV < 100 and ≥ 100

EBV	Diagnosed with IVDH	IVDH clinical signs	Free of clinical signs	Total
< 100	21	5	24	50
≥ 100	2	1	64	67
Total	23	6	88	117

### Genotyping

All 151 Dachshunds (84 wire-haired, 32 smooth-haired and 35 long-haired) were genotyped for the *Ins* allele. Genotypes and χ2 values are presented in Table 4.

**Table 4:** Genotyping of the *Ins* allele in wire-, long- and smooth-haired Dachshunds with ≥5 and < 5 calcifications

Number of calcifications	***Ins/Ins***	***Ins/N***	***N/N***	Total	Χ^**2**^	***p***-value
**Wire-haired**					37.13	< 0.0001
≥ 5	32	1	0	33		
< 5	15	29	7	51		
Total				84		
**Long-haired**					0.22	0.89
≥ 5	13	0	0	13		
< 5	22	0	0	22		
Total				35		
**Smooth-haired**					3.75	0.15
≥ 5	17	0	0	17		
< 5	12	2	1	15		
Total				32		

*Ins* designates the mutated allele (i.e. the *FGF4* retrogene insertion); *N* designates the normal allele (i.e. no insertion).

In the wire-haired Dachshunds the frequency of the *Ins* allele was 0.74 and the genotype and calcification status were significantly associated (*p* < 0.0001). The test sensitivity was (32 + 1)/33 = 1 and the test specificity was 7/51 = 0.14.

In long- and smooth-haired Dachshunds the *Ins* allele was fixed or almost fixed with no association with calcification status (*p* = 0.89 and 0.15 respectively).

## Discussion

Since the effective population size is quite small in most dog breeds it is very important to evaluate both breeding goals and breeding schemes carefully. IVDH is a painful and also highly heritable condition which has to be taken into account in breeding schemes in breeds with a high incidence of the disease. It is, however, important to use a breeding scheme that ensures that the genetic variation is maintained in the population.

The incidence of IVDH is not significantly different between the three hair variants in Dachshunds. Considering an overall incidence of 18% and the fact that one third of these dogs are euthanized due to this condition, IVDH is a significant health problem in this breed. If dogs with IVDH clinical signs are included the average incidence is 26%. The questionnaires are based on different veterinarians’ diagnoses (potentially based on slightly different criteria) and owners’ evaluations. However, considering the sample size (503) the estimated incidence is reliable. This is supported by the fact that our findings are in concordance with incidence estimates found in other studies [3–5].

The follow-up study confirms previous studies [4, 15] showing that disc calcification and IVDH are highly associated. Dachshunds with ≥5 calcifications have a 14 times higher risk of developing IVDH than dachshunds with < 5 calcifications. Among dogs with 5 calcifications (*n* = 12) half were diagnosed with IVDH and half were free of clinical signs. Among dogs with ≥6 calcifications (*n* = 19) 13 had been diagnosed with IVDH, 4 had shown clinical signs of IVDH and only 2 had been free of clinical signs (see Additional file 2). In the low-risk group (< 5 calcifications) 80/86 were free of clinical signs. These figures confirm that the number of disc calcifications (≥ 5 or < 5) at the age of 2–4 years is a valid risk indicator for IVDH.

For dogs with an EBV < 100 the risk of developing IVDH is 15 times higher than for dogs with EBV ≥ 100. This further emphasizes that calcification status is a valid risk indicator and confirms the high heritability of this trait.

The genotyping results show that overall, the frequency of the *Ins* allele is very high. This is in accordance with the results reported by Batcher et al. [19]. However, we show that there is a clear difference between hair variants. That is, all long-haired Dachshunds were homozygous for the insertion and among the smooth-haired only one was *N/N* and two were *Ins/N*. According to Batcher et al. [19] and the UC Davis Veterinary Genetics Laboratory homepage [6] the insertion affects the risk of IVDH in a dominant manner, and dogs with at least one copy of the insertion is at risk of developing disc herniation. Thus, using the insertion genotype as selection criteria all the long-haired and almost all (31/32) of the smooth-haired Dachshunds would be excluded from breeding.

Among the wire-haired dogs the insertion genotype is significantly associated with calcification status. Assuming that the insertion has a dominant effect on the risk of IVDH both *Ins*/*Ins* and *Ins*/*N* are at risk. Obviously, the high sensitivity of 1.0 is owing to the overall high *Ins* allele frequency.

The specificity of 0.14 implies that 86% of the dogs with low risk of IVDH would be excluded and only 7/84 or 8% of the wire-haired dogs would be left suitable for breeding according to the DNA test. This would have a detrimental impact on the genetic variation in this population. In comparison, radiographic back examination has a sensitivity of 0.79. Thus 21% of the dogs who develop back problems have only few calcifications and are therefore not excluded from breeding when using this method. However, IVDH is a multifactorial disease and other genetic components and environmental factors may dispose some dogs with few calcifications to IVDH. In contrast to the DNA test, the specificity of the radiographic back examination is high (0.91) since only 9% of the dogs who are excluded from breeding are presumed to remain healthy. Although not perfect, the present breeding scheme relying on calcification scores is more accurate than the DNA test.

In a validation study of the candidate region found on CFA12 Mogensen et al. [17] genotyped 3 SNPs in protein coding regions/untranslated region (UTR) and found significant association with calcification status in the wire-haired study population. A sample of smooth- and long-haired cases and controls were also genotyped and found fixed for the disease associated haplotype. This is in line with our genotyping results showing that these two hair variants are almost fixed for the *Ins* allele associated with disease in the wire-haired dogs. Thus, it cannot be ruled out that other elements within the region on CFA12 have an impact on the development of IVDD.

In the IVDD GWAS performed by Brown et al. [18] no distinction was made between hair variants. Moreover, except for Coton de Tuléar the breeds in the case group are not matched in the control group. In another study [19] the overall frequency of the *Ins* allele has been estimated for most of the breeds included in the IVDD GWAS performed by Brown et al. [18]. It is evident that the breeds used in the case group in general have a high *Ins* allele frequency (for 6/9 of the case breeds the allele frequency is ≥0.57), and that the breeds used in the control group in general have a low *Ins* allele frequency (for at least 4/8 control breeds the allele frequency ≤ 0.08). The association found may therefore be caused by a breed effect reflecting an overall difference in allele frequencies. On the other hand, the same region on CFA12 has been identified by Mogensen et al. [8] in a GWAS using only wire-haired Dachshund cases and controls. This supports that this region harbors genetic components influencing the risk of developing disc herniation. Future studies are, however, needed to fully unravel the genetic contribution to IVDH.

## Conclusion

Our results show that the *FGF4* retrogene insertion on CFA12 is not a valid risk indicator on its own in the Dachshund populations. Relying on the DNA test will have an irreversible effect on the Dachshund breed excluding almost all dogs from breeding. Thus, using calcification status remains the most reliable breeding scheme for disc herniation.

## Supplementary Information

**Additional file 1.** Questionnaire. Letter to dog owners (follow-up study), letter to dog owners (incidence study) and questionnaire.

**Additional file 2.** Calcification groups. The number of dogs in each calcification group (0–12) and their back status.

## Data Availability

All data analyzed in the incidence and follow-up studies are included in this published article and its supplementary information files. Genotyping gel pictures are available from the corresponding author on reasonable request.

## Abbreviations

BLUP: Best linear unbiased prediction
CFA: *Canis familiaris* (chromosome)
EBV: Estimated breeding value
EDTA: Ethylene-diamine-tetra-acetic
FGF4: Fibroblast growth factor 4
GWAS: Genome wide association study
Ins: Designates the fibroblast growth factor 4 retrogene insertion allele
IVDD: Intervertebral disc disease
IVDH: Intervertebral disc herniation
N: Designates the normal allele (i.e. no insertion of the fibroblast growth factor 4 retrogene)
OR: Odds ratio
RR: Relative risk
SNP: Single nucleotide polymorphism
UTR: Untranslated region
