# Supplementary material for: Breeding schemes for intervertebral disc disease in dachshunds: Is disc calcification score preferable to genotyping of the FGF4 retrogene insertion on CFA12?
Source: Canine Med Genet. 2020 Dec 1;7:18. doi: 10.1186/s40575-020-00096-6 (PMC7708201; doi:10.1186/s40575-020-00096-6)
Supplement: Supplementary file 1 — Additional file 1. Questionnaire. Letter to dog owners (follow-up study), letter to dog owners (incidence study) and questionnaire. [file 40575_2020_96_MOESM1_ESM.docx]

**Additional file 1: Questionnaire**

Letter to dog owners: incidence study

Dear Dachshund owner,

We hope you will take the time answering the attached questionnaire. The survey is an important part of a master thesis, which will hopefully contribute important new knowledge on disc herniation in Dachshunds.

In our master thesis we will investigate the incidence of back problems in Dachshunds of all hair coat and size variants. Both the Danish Kennel Club and the Danish Dachshund Club have a strong interest in having such an investigation carried out.

Being the owner of a Dachshund you may be aware that disc herniation is a known problem in Dachshunds. Various factors influence the development of this disease including genetics. Therefore, radiographic examination of the back has been a requirement for the dog to be used for breeding. This requirement has now been changed to a recommendation.

In 2001 – 12 years ago - a master thesis was done to survey the incidence of disc herniation in wire-haired standard size Dachshunds. It would therefore be interesting to perform a comparable study to show the development of the incidence. Thus, this survey is a follow-up on the master thesis from 2001 but including all hair coat and size variants.

Your dog has been randomly chosen among all DKC registered Dachshunds born in 2002-2004. All information about your dog will be kept confidential and all data will appear as numbers without name, stud book number or any other type of identification.

If the dog has moved to a new owner, it would be very helpful, if you can contribute with information so that we can contact the new owner.

We would be grateful if you return the questionnaire as soon as possible and no later than February 10, 2014. The enclosed, stamped envelope can be used.

Please do not hesitate to contact us if you have any questions about the thesis, questionnaire or other.

Thank you in advance!

Best regards

Tine Marx Allentoft (phone no. xx xxxxxx) and Charlotte Mørch Andersen (phone no. xx xxxxxx)

*The letter has been translated from Danish.*

Letter to dog owners: follow-up study

Dear Dachshund owner,

We hope you will take the time answering the attached questionnaire. The survey is an important part of a master thesis, which will hopefully contribute important new knowledge on disc herniation in Dachshunds.

In our master thesis we will investigate the incidence of back problems in dachshunds of all hair coat and size variants. Moreover, we will evaluate the association between the result of the radiographic examination and the development of disc herniation. Both the Danish Kennel Club and the Danish Dachshund Club have a strong interest in having such an investigation carried out.

Being the owner of a Dachshund you may be aware that disc herniation is a known problem in Dachshunds. Various factors influence the development of this disease including genetics. Therefore, radiographic examination of the back has been a requirement for the dog to be used for breeding. This requirement has now been changed to a recommendation.

All dogs radiographically examined in 2004-2006 have been chosen for this investigation, and therefore we contact you as the owner of:

All information about your dog will be kept confidential and all data will appear as numbers without name, stud book number or any other type of identification.

If the dog has moved to a new owner, it would be very helpful, if you can contribute with information so that we can contact the new owner.

We would be grateful if you return the questionnaire as soon as possible and no later than February 10, 2014. The enclosed, stamped envelope can be used.

Please do not hesitate to contact us if you have any questions about the thesis, questionnaire or other.

Thank you in advance!

Best regards

Tine Marx Allentoft (phone no. xx xxxxxx) and Charlotte Mørch Andersen (phone no. xx xxxxxx)

*The letter has been translated from Danish.*

Questionnaire (incidence and follow-up study)

Name:

Stud book number:

Hair coat variant:

Date:

1. Is your dog still alive?

If it is dead, what was the cause:

Disc herniation □ Unknown □ Other □

Other causes may be noted here:

How old was your dog when it died? (e.g. 7 years and 2 months)

2. Has your dog had any of the following symptoms: Yes □ No □

If yes, which?

Stiff back □

Curved back □

Lameness (one or more legs) □

Reluctance to walk □

Reluctance to jump □

Difficulties getting up □

Pain when lifted □

Pain at touching □

Hind limb paresis □

Lurching □

Reduced mobility □

Paraesthesia (hind quarters) □

Difficulties defecating □

Difficulties urinating □

Other □

When did these symptoms occur? (This may be hard to remember precisely but please note an approximate age):

Have the symptoms been recurrent? Yes □ No □

Did a veterinarian examine your dog due to these symptoms? Yes □ No □

In that case - what was the diagnosis?

3. Has a veterinarian ever diagnosed your dog with disc herniation? Yes □ No □

If yes, how old was the dog when it was diagnosed?

**Thank you for taking your time answering the questionnaire.**

*The questionnaire has been translated from Danish and questions not included in this article have been omitted.*
