# Supplementary material for: Breeding schemes for intervertebral disc disease in dachshunds: Is disc calcification score preferable to genotyping of the FGF4 retrogene insertion on CFA12?
Source: Canine Med Genet. 2020 Dec 1;7:18. doi: 10.1186/s40575-020-00096-6 (PMC7708201; doi:10.1186/s40575-020-00096-6)
Supplement: Supplementary file 2 — Additional file 2. Calcification groups. The number of dogs in each calcification group (0–12) and their back status. [file 40575_2020_96_MOESM2_ESM.docx]

**Additional file 2: Calcification groups**

| **Number of calcifications** | **Diagnosed with disc herniation** | **Back pain** | **Free of symptoms** | **Total** |
| --- | --- | --- | --- | --- |
| **0** | 0 (0,0 %) | 0 (0,0 %) | 16 (13,68 %) | 16 (13,68 %) |
| **1** | 1 (0,85 %) | 1 (0,85 %) | 22 (18,80 %) | 24 (20,51 %) |
| **2** | 1 (0,85 %) | 0 (0,0 %) | 17 (14,53 %) | 18 (15,38 %) |
| **3** | 2 (1,71 %) | 0 (0,0 %) | 13 (11,11 %) | 15 (12,82 %) |
| **4** | 0 (0,0 %) | 1 (0,85 %) | 12 (10,26 %) | 13 (11,11 %) |
| **5** | 6 (5,13 %) | 0 (0,0 %) | 6 (5,13 %) | 12 (10,26 %) |
| **6** | 2 (1,71 %) | 0 (0,0 %) | 0 (0,0 %) | 2 (1,71 %) |
| **7** | 1 (0,85 %) | 1 (0,85 %) | 2 (1,71 %) | 4 (3,42 %) |
| **8** | 4 (3,42 %) | 1 (0,85 %) | 0 (0,0 %) | 5 (4,27 %) |
| **9** | 1 (0,85 %) | 1 (0,85 %) | 0 (0,0 %) | 2 (1,71 %) |
| **10** | 3 (2,56 %) | 0 (0,0 %) | 0 (0,0 %) | 3 (2,56 %) |
| **11** | 1 (0,85 %) | 0 (0,0 %) | 0 (0,0 %) | 1 (0,85 %) |
| **12** | 1 (0,85 %) | 1 (0,85 %) | 0 (0,0 %) | 2 (1,71 %) |
| **Total** | **23 (19,66 %)** | **6 (5,13 %)** | **88 (75,21 %)** | **117 (100 %)** |
